# The impact of plate length, fibula integrity and plate placement on tibial shaft fixation stability: a finite element study

**DOI:** 10.1186/s13018-019-1088-y

**Published:** 2019-02-15

**Authors:** Yasen Cao, Yong Zhang, Lixin Huang, Xiaowei Huang

**Affiliations:** The First Affiliated Hospital of Soochow University, 188, Shi Zi Road, Suzhou, 215006 China

**Keywords:** Finite element analysis, Tibial shaft fracture, Peak stress, Displacement, Plate length, Fibula integrity, Placement of plate

## Abstract

**Background:**

Tibial shaft fractures account for approximately 15% of long bone fractures. Locked plates with minimally invasive plate osteosynthesis techniques are used widely by surgeons. The purpose of this study is to investigate the impact of factors meaning the plate length, fibula integrity, and placement of the plate on the stability of tibial shaft fracture fixation.

**Methods:**

A finite element model of the tibial shaft fracture was built. An axial force of 2500 N was applied to simulate the axial compressive load on an adult knee during single-limb stance. The equivalent von Mises stress and displacement of the fractured ends were used as the output measures.

**Results:**

In models with plates on the lateral side of the tibia, displacement in models fixed with a 12-hole plate showed the smallest value. In models with plates on the medial side of the tibia, displacement in models fixed with 14-hole plate showed the smallest value. The peak stress of plates implanted on the medial side of the tibia was higher than that of plates on the lateral side. The peak stress and the displacement of models involved with the fibula were lower than that of models without fibula, regardless of the length or location of plates.

**Conclusions:**

For models with plates on the medial side of the tibia, the 14-hole plate is the best choice in terms of stability. While for models with plates on the lateral side of the tibia, the 12-hole plate demonstrated the optimal biomechanical stability. The integrity of the fibula improves the anti-vertical compression stability of the construct. The peak stress of plates implanted on the medial side of the tibia was higher than that of plates on the lateral side, which indicated that the construct with medially implanted plate has a higher risk of implant failure.

## Background

Tibial shaft fractures account for approximately 15% of long bone fractures which usually occur as a result of road accidents caused by high-energy trauma [1]. In this case, surgical approaches are often involved to maintain anatomical reduction and to prevent the development of devastating complications.

To fix the fractured tibial shaft, various approaches have been employed. Plate fixation with minimally invasive plate osteosynthesis (MIPO) techniques and locked intramedullary nailing are the two most frequently used surgical approaches [2]. Although locked intramedullary nailing is the golden standard for treating fractures of the tibial shaft [3], the locking plate-screw fixation is also widely used and proved to be an effective option, especially when fracture line is extending to metaphysis [4, 5]. In addition, the MIPO technique enables limited dissection of surrounding soft tissues which help to preserve blood supply and fracture hematoma at the fracture site and thus promoting biological bone healing [6]. According to a newly published meta-analysis, neither technique shows a clear advantage regarding the risk of malunion/non-union or functional outcome [5]. Like other plate fixations, there are many factors that can influence the mechanical stability of percutaneous plate fixation, such as plate length, fibula integrity, and placement of the plate. However, there are few biomechanical studies focusing on this topic.

Finite element analysis is one of the computational methods that have received wide acceptance in the field of orthopedic research, where three-dimensional models of bone-implant construct are converted into finite elements with the application of simulated physiological loads to analyze and predict the outcome of surgery [7, 8]. Keyak et al. have used finite element methods to build a femur bone model with the intention to predict femoral fracture loading patterns [9]. Teo and Ng have published a study concentrating on injure mechanisms and stress distribution patterns of human atlas using this technique [10]. Biomechanical studies via computational simulation can provide deeper insight into the stability and functionality of bone constructs [8]. As a consequence, in the present study, we intended to investigate the impact of the factors on the stability of plate fixation on tibial shaft fractures using the finite element method.

## Materials and methods

### The establishment of three-dimensional models

The present work was approved by the Ethics Committee of the local institute and was performed in accordance with the Declaration of Helsinki.

**Fig. 1:**
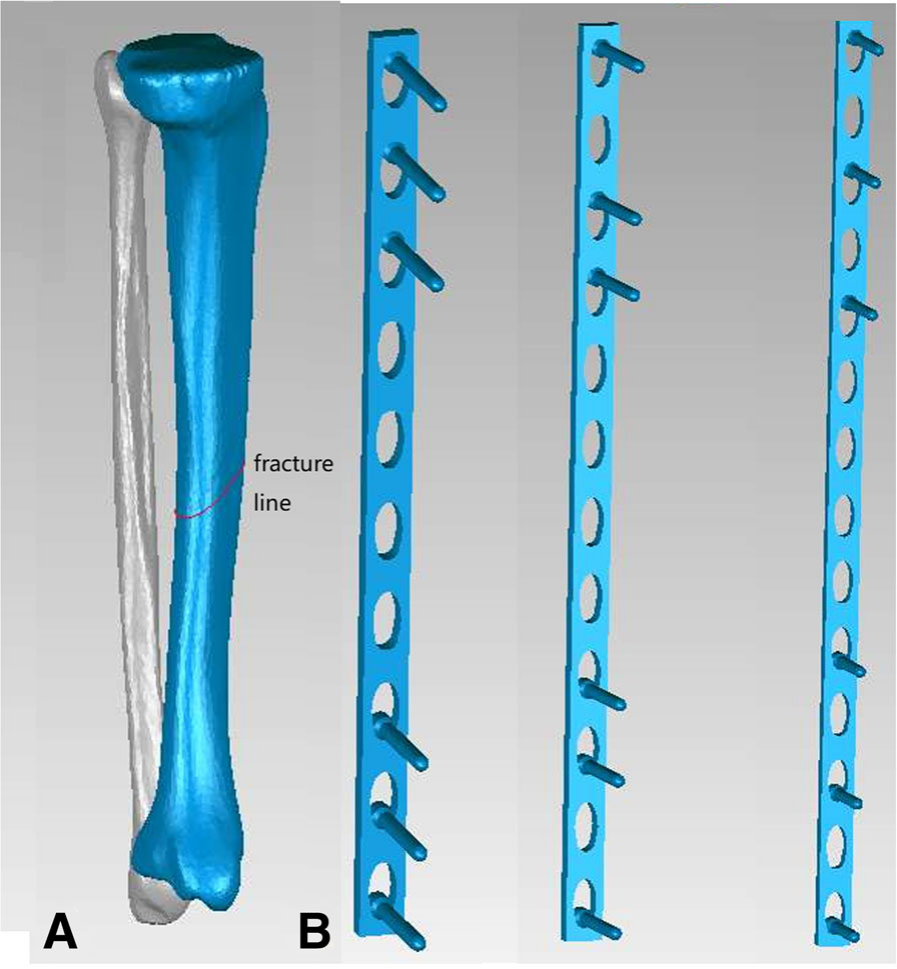
**a** The tibial shaft fracture model. **b** The different plates (10, 12, and 14 holes) and the placement of the screws

**Fig. 2:**
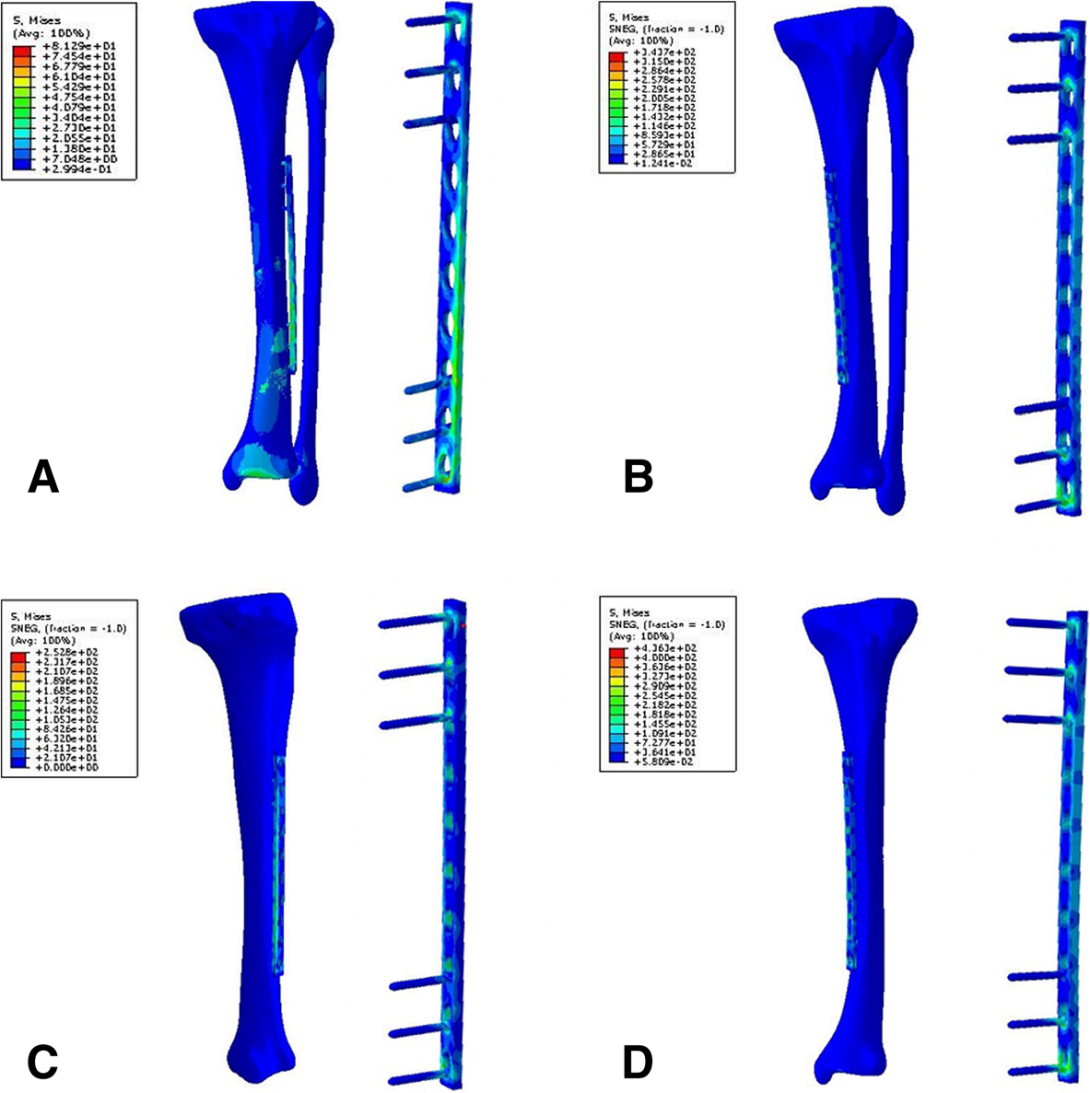
The stress distribution of models. **a** P10TFLa. **b** P10TFMa. **c** P10TLa. **d** P10TMa

**Fig. 3:**
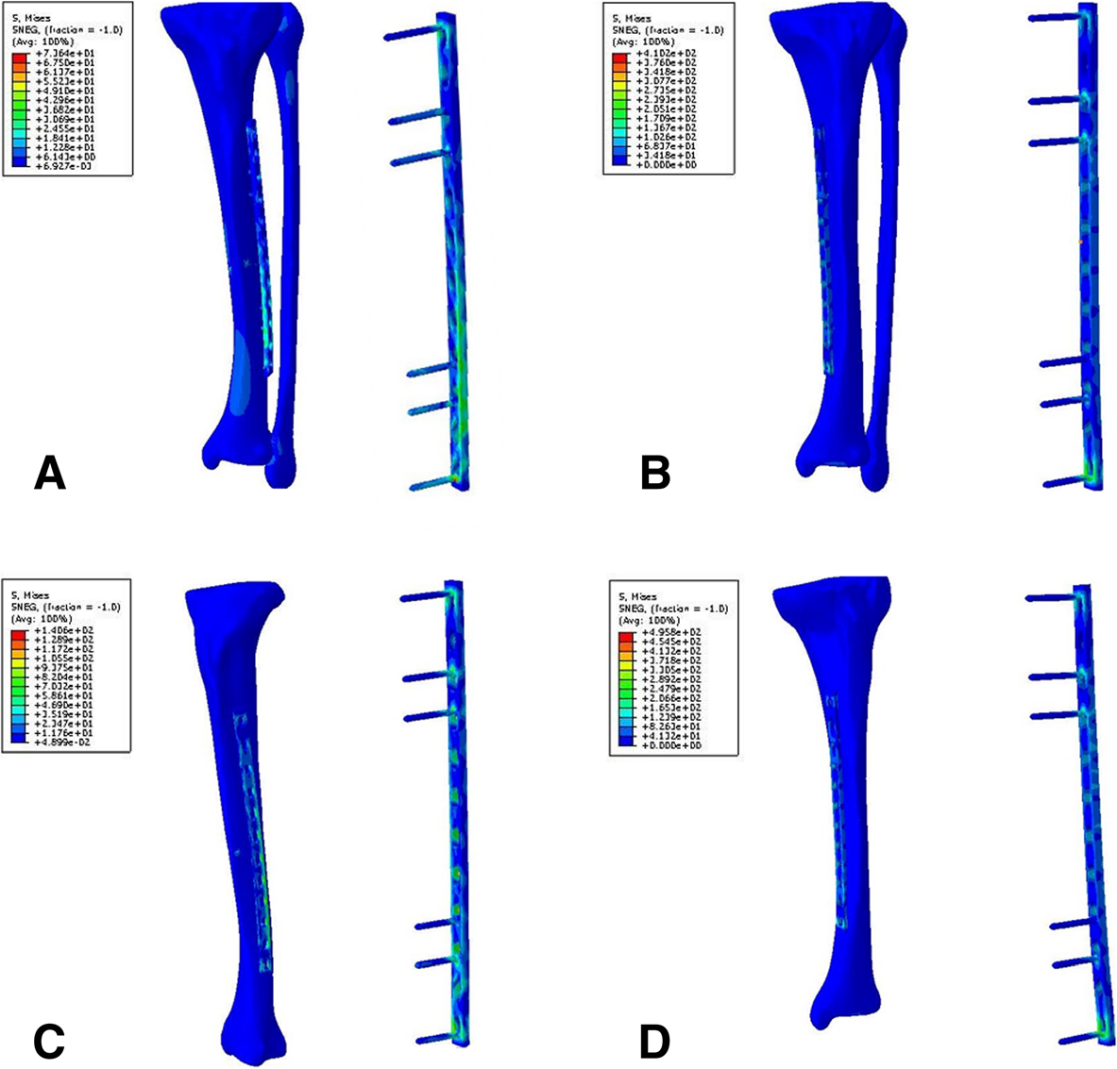
The stress distribution of models. **a** P12TFLa. **b** P12TFMa. **c** P12TLa. **d** P12TMa

**Fig. 4:**
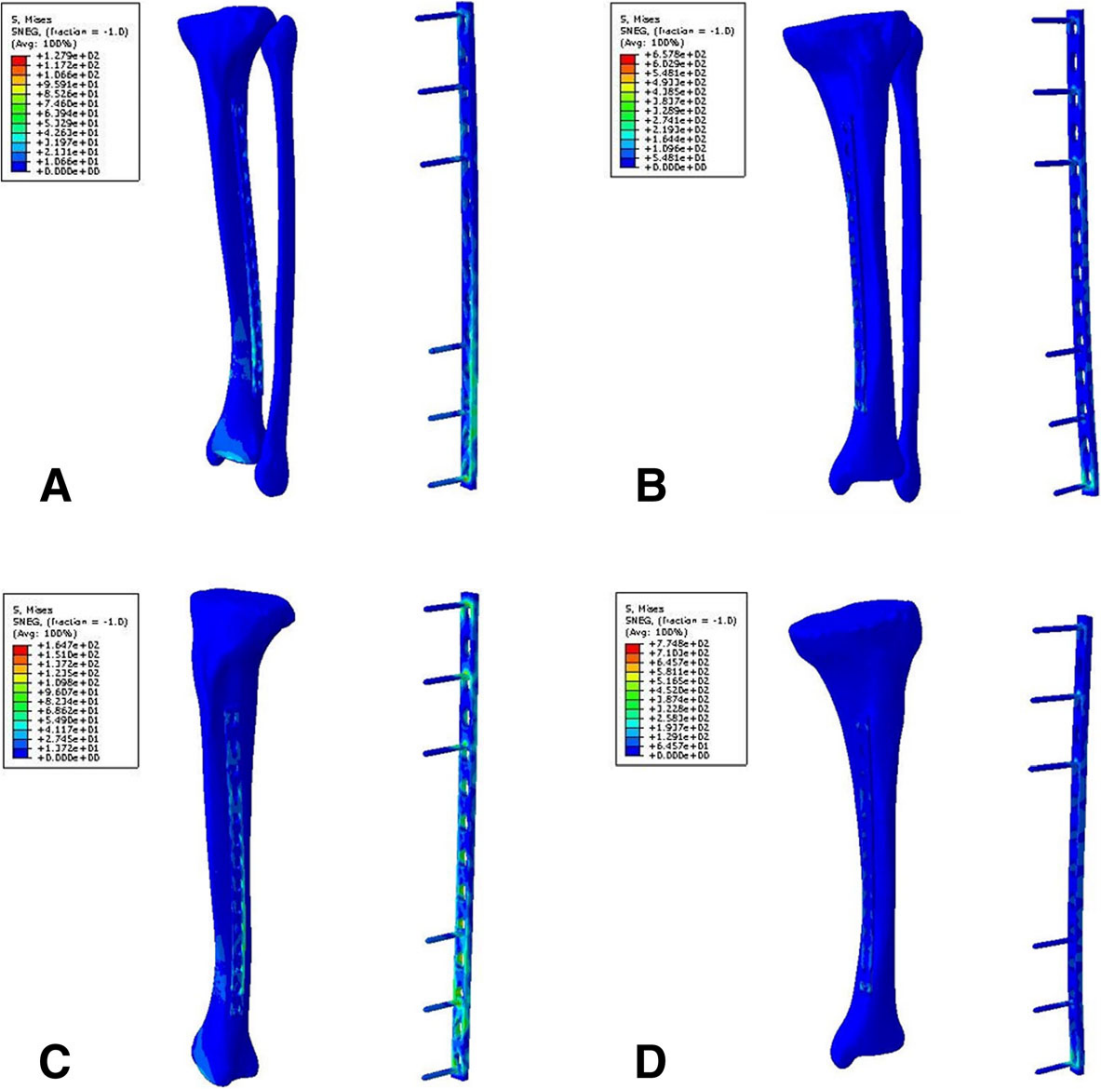
The stress distribution of models. **a** P14TFLa. **b** P14TFMa. **c** P14TLa. **d** P14TMa

CT scan images of the right leg were extracted from a 35-year-old healthy male, 175 cm in height and 70 kg in body weight. No anatomical abnormality in his right leg was found. The CT scan data was taken with 1-mm intervals from plane 10 cm above the knee down to the plane 5 cm under the ankle. CT data in DICOM form were then imported into Mimics 11.5 software (Materialize, Leuven, Belgium) to reconstruct the geometrical surface of the tibia and fibula. The 3D structure of the tibia and fibula saved as STL format was then imported to Geomagics Studio 12.0(Raindrop Company, USA), where procedures were performed to smooth the surface of the models and delete the protruding triangles and exported to Hypermesh 11.0 (Altair Engineering, Inc., USA). To simulate transverse fracture of the tibia, an oblique fracture line with 45° angulations was made in the middle of the tibia shaft, as demonstrated in Fig. 1a. To illustrate the perfectly fitted fracture model, there was no fracture gap between the two fragments. In our study, we compared the different length of the plate (10 holes, 12 holes, and 14 holes) fixed with 6 screws, with 3 screws placed in each side of the fracture line. The placement of screws was made to ensure that the working length of all plates equals, as shown in Fig. 1b. In addition, models with or without fibula as well as two different placement patterns are also constructed to study the impact of fibula integrity or plate placement on construct stability. Overall, 12 different fixation modalities were constructed, as shown in Figs. 2, 3, and 4. The 3D models of the implants were designed according to the description in Hu et al. using computer-aided design (CAD) software (SolidWorks 2012, DS SolidWorks Corp., USA). All the models were pre-processed using the software HyperMesh 11.0 (Altair Engineering, Inc., USA) and then exported to Abaqus 6.14 (Simulia Corp., USA) in the format of INP for calculation.

### Finite element modeling

In our study, the mechanical properties of the bones and implants were assumed to behave as homogeneous, isotropic, and linearly elastic material and adopted from previous published reports [7, 8, 11]. The contact pair of fractured surfaces was assigned with the coefficient of friction 0.003 [12] and that of bone-screw implant was assumed to be tough. The tibia-fibula interface was tied. As for boundary conditions, the distal end of the tibia was fixed and axial forces of 2500 N with a distribution of 60% to the medial compartment were utilized to simulate the axial compressive load on an adult knee [8, 13].

### Nomenclature

In this study, the tibia and fibula were designated as “T” and “F,” respectively. P was the abbreviation for the plate. Lateral and medial plating were designated as “L” and “M.” The application of an axial load was designated as “a.” For example, “P10” means the plate has 10 screw holes and a plate of 10 holes implanted on the lateral side of the tibia with an intact fibula was labeled as “P10TFL.” Similarly, the application of an axial compressive load applied to the model was labeled as “P10TFLa.”

## Result

### Peak stresses of plates fixed with plates of different length

**Fig. 5:**
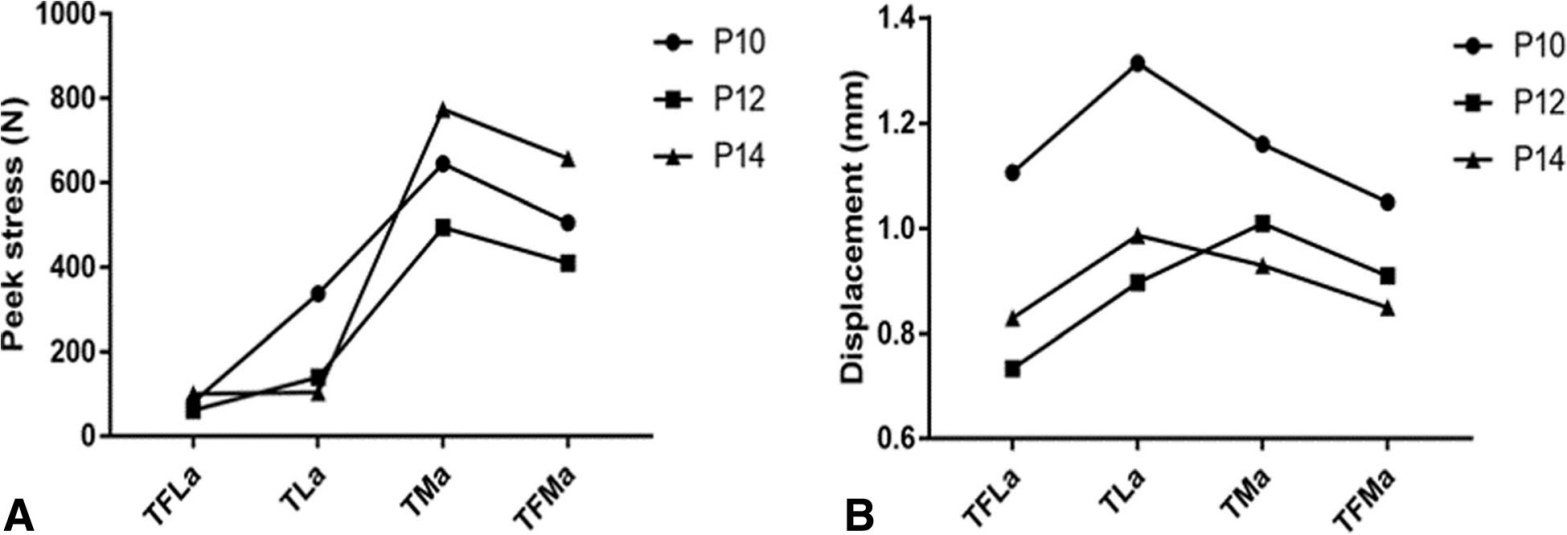
**a** Peak stresses on the plates with different length. **b** Fractured end displacement fixed with plates of different length

The effect of plate length on the peak stress in the models with axial load was analyzed, as shown in Fig. 5. The peak stress of P14 in P14TFLa is higher than that of P10 in P10TFLa. The peak stress of P10 in P10TFLa was higher than that of P12 in P12TFLa. While in the model without fibula, the peak stress value of P10 was the highest and that of P14 was the lowest.

For plates on the medial side of the tibia, the peak stress of the P14 was higher than that of the P10 and the peak stress value of P12 was lower than that of P10, regardless of the integrity of the fibula, as shown in Fig. 5a.

### Displacement of fractured ends fixed with plates of different length

The effect of plate length on fractured end displacement in the models with axial load was analyzed, as shown in Fig. 5b. In models with plates implanted on the lateral side of the tibia, the displacement in models fixed with P10 was the highest while that in models fixed with P12 showed the smallest value. In models with plates implanted on the medial side of the tibia, the displacement in models fixed with P10 was the highest as well while that in models fixed with P14 showed the smallest value.

### Fractured end displacement of models with plates on the medial or lateral side

**Fig. 6:**
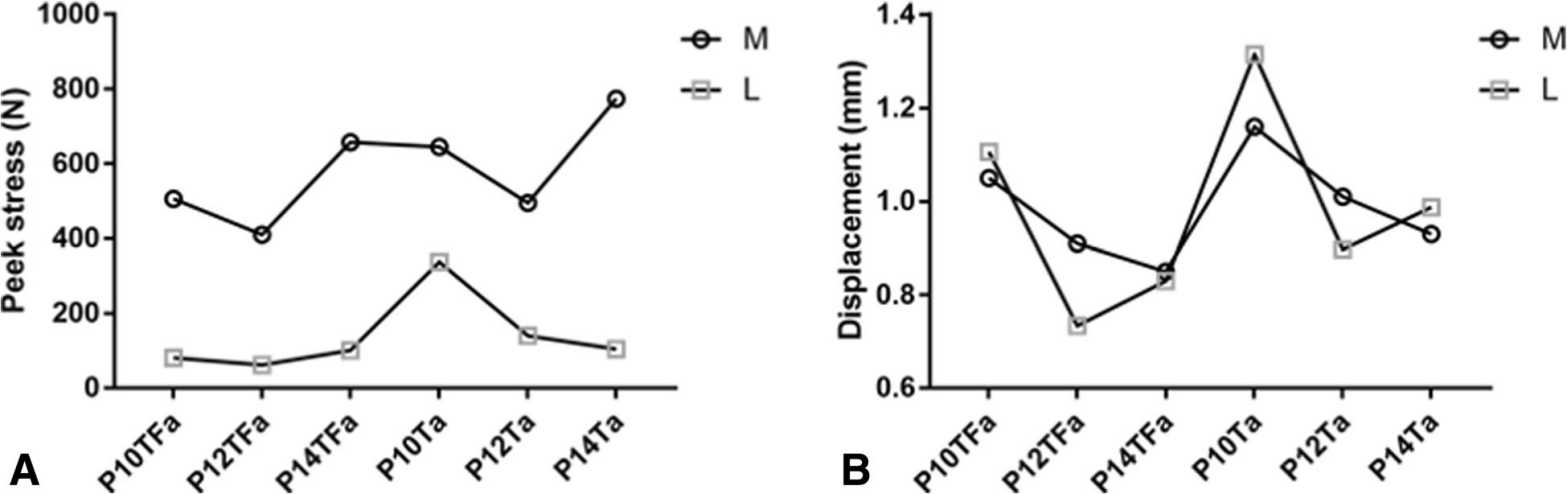
**a** Peak stresses of plates on the medial or lateral side. **b** Fractured end displacement of models with plates on the medial or lateral side

The effect of plating location on the displacement of fracture end in the models with axial load was analyzed, as shown in Fig. 6a. For P10TFa, P10Ta, and P14Ta, the fracture end displacement is higher in models fixed with plates on the lateral side of the tibia. However, the remaining models showed the opposite pattern.

### Stress distribution of plate on the medial or lateral side

The effect of plate location on the peak stress of plates in the models with axial load was analyzed, as shown in Fig. 6b. It was found that the peak stress of plates implanted on the medial side of the tibia was higher than that of plate stress on the lateral side. For plates fixed on the medial side of the tibia, stress concentrated on the intersection between the most distal screw and the plate hole. But when the plates fixed on the lateral side of the tibia, stress also concentrated on the distal part of the plate, as shown in Fig. 2, 3, and 4.

### Importance of the integrity of fibula

**Fig. 7:**
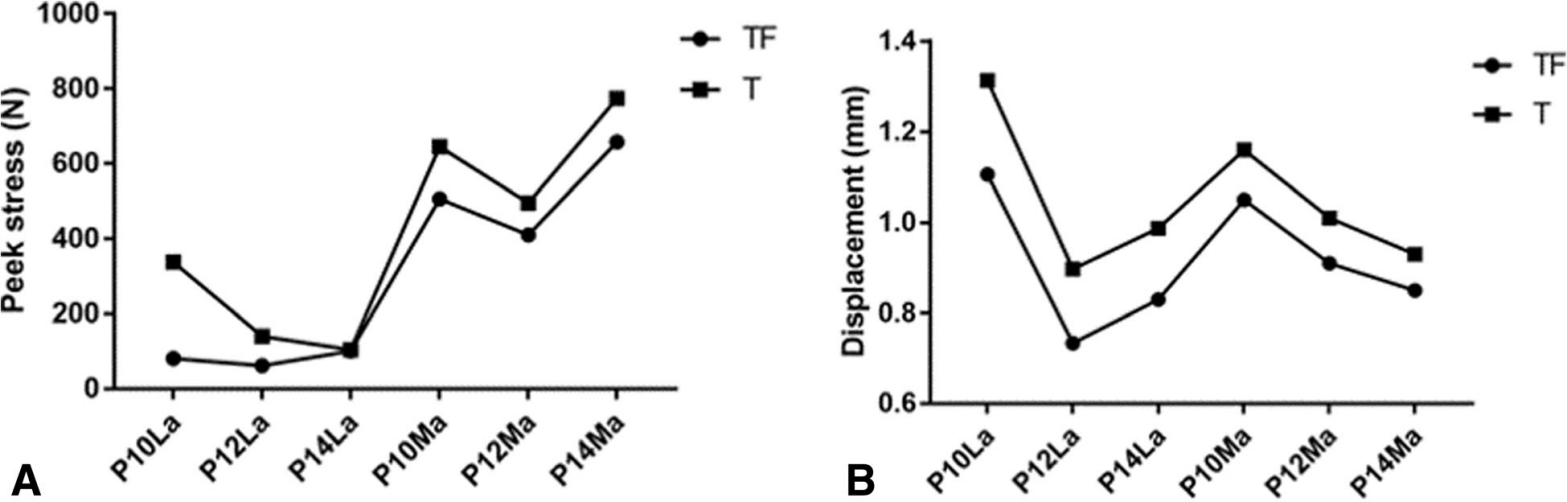
**a** Peak stress of plates in models with or without fibula. **b** Fractured end displacement of models with fibula or without fibula

The effect of integrity of the fibula on the peak stress in the models with axial compression load was compared, as shown in Fig. 7. The peak stress of models involved with the fibula was lower than that of models without fibula, regardless of the length or location of plates, as shown in Fig. 7a.

The effect of integrity of the fibula on the fracture end displacement in the models with axial load was analyzed, as shown in Fig. 7b. The displacement of models involved with the fibula was lower than that of models without fibula, regardless of the length or location of plates.

## Discussion

### Models and screw placement

Our study was designed to help understand what factors affect tibial fracture treated with plate fixation and how to choose an optimal plate fixation modality, which may facilitate our clinical decision-making process. In this study, all plates were fixed with 6 screws, with 3 screws placed in each side of the fracture line. We had learned from the research of Stoffel et al. and Leung et al. that more than three screws per fragment did little to increase axial stiffness [14, 15]. Stoffel et al. had reported that axial stiffness is mainly influenced by the working length of the plate construct (working length means the distance between the first screw at each side of the fracture) [14]. As a result, in this study, we make sure that all plates share the same working length, in order to eliminate the impact of working length. The gap of two fractured surface significantly affects construct stability. Oh et al. had reported that even a thin fracture gap (1 mm) with no contact between the fracture after plating decreases stiffness exponentially [16, 17]. As a result, in this study, no fracture gap between the two fracture ends was made.

### The effect of plate length on construct stability

We found that in models where plates were implanted on the medial side of the tibia, displacement in models fixed with P10 was the highest while that of P14 showed the smallest value, which indicates that construct rigidity is increased when the length of the plate is prolonged. This is in accordance with several previously published clinical researches which concluded that the use of relatively longer plates is a vital technical factor that can reduce the risk for fixation failure [18–20].

For models with implants on the lateral side of the tibia, the displacement in models fixed with P10 was the highest while that of models fixed with P12 showed the smallest value, which indicates 12-hole plate had an advantage over the 10-hole plate as well as the 14-hole plate in the construct rigidity. This result was partly consistent with the hypothesis mentioned above. Namely, relatively longer plates (P12, P14) are better than a short plate (P10). However, P12, not P14, was best for models with implants on the lateral side of the tibia, which may indicate that for tibial fractures fixed with a lateral plate, there existed an optimal plate length for construct stability.

### The location of the plate, medial, or lateral

Although Shon and Park had reported that both medial and lateral MIPO for treating distal tibial fractures produced good clinical and radiological results [21], we wanted to know which is better in biomechanical testing. In the present study, it was found that the peak stress of plates implanted on the medial side of the tibia was higher than that of plates on the lateral side. This can be explained that the medial side of the tibia is the tension side and medially placed plate consequently undertakes more pressure [8, 13]. Choosing a medical tibia plating means an easier operation approach than plating on the lateral side of the tibia because of the thin soft tissue of the medial tibia, which can shorten the time of operation [22]. However, the higher peak stress on medially implanted plate may lead to a higher risk of implant failure, especially when patients are overweight [13].

### The contribution of the fibula (fibula or without fibula)

As shown in Fig. 7, under an axial load of 2500 N, the peak stress of models involved with the fibula in all groups was lower than that of models without fibula, regardless of the length or location of plates. And the displacement of fracture end of models with an intact fibula was lower than that of models without fibula, regardless of the length or location of plates, which indicated that the integrity of the fibula had a positive impact on the stability of the construct. There were many reports published studied the role of fibular fixation in shaft fractures of the leg. Goh et al. had reported that load transmission through the fibula varied with ankle position. With the ankle at the neutral position, the load distribution to the fibula averaged 7.12% of the total force transmitted through the tibia and fibula [23]. Weber et al. had reported that plating the fibula can decrease motion across a tibial defect when the fixation was less rigid [24].

### Limitation

There are a few limitations inherent in our study. First, the fracture models were simplified and idealized, the materials of the cortical and cancellous bone were both imitated, and the soft tissue was excluded. The actual conditions of bone properties were more complicated and cannot be reflected perfectly. Second, cyclic loading was not simulated because the simulation of the dynamic motion of the joints is time-consuming and requires substantial computer resources [8]. Thus, the displacement calculated may be underestimated. Finally, the tibiofibular joints were simplified and simulated by bonding the tibia and fibula together, which may not reflect the condition of the actual joint motion.

## Conclusion

For models with plates implanted on the medial side of the tibia, P14 is the best choice in terms of stability. While for models with plates implanted on the lateral side of the tibia, P12 demonstrated the optimal biomechanical stability. The integrity of the fibula has a positive impact on the anti-vertical compression stability. The peak stress of plates implanted on the medial side of the tibia was higher than that of plates on the lateral side, which indicated that the construct with medially implanted plate has a higher risk of implant failure.

## Abbreviations

DICOM: Digital Imaging and Communications in Medicine
MIPO: Minimally invasive plate osteosynthesis
